# Working With AI and Robotic Systems While Preserving Clinical Judgement, Professional Accountability and Person‐Centred Care: Critical Care Nurses’ Perspectives

**DOI:** 10.1111/nicc.70695

**Published:** 2026-10-12

**Authors:** Tuba Sengul, Dilek Yilmaz Akyaz, Buse Macit Kaplan, Yunus Emre Akyol, Tuğba Cevizci, Melek Demiral, Deema Mahasneh, Noordeen Shoqirat, Holly Kirkland‐Kyhn

**Affiliations:** ^1^ Koç University School of Nursing Istanbul Türkiye; ^2^ Graduate School of Health Sciences Koç University Istanbul Türkiye; ^3^ Koç University Hospital Istanbul Türkiye; ^4^ Haydarpaşa Numune Training and Research Hospital University of Health Sciences Istanbul Türkiye; ^5^ Nursing Department, College of Pharmacy and Health Sciences Ajman University Ajman UAE; ^6^ Faculty of Nursing Mutah University Karak Jordan; ^7^ Faculty of Health Sciences Higher Colleges of Technology Ras Al Khaimah UAE; ^8^ Betty Irene Moore School of Nursing University of California Davis USA

**Keywords:** artificial intelligence, clinical decision‐making, critical care nursing, person‐centred care, robotics

## Abstract

**Background:**

The integration of AI and robotic systems into critical care necessitates examining how ICU nurses preserve clinical judgement, professional accountability and person‐centred care while using these technologies.

**Aims:**

To explore ICU nurses' perceptions of AI and robotic systems in critical care, focusing on clinical judgement, trust, responsibility, professional roles and person‐centred care.

**Study Design:**

A qualitative descriptive study using scenario‐based elicitation was conducted. Data were collected through seven semi‐structured focus group discussions (FGDs) with 38 ICU nurses working in general, paediatric and cardiovascular surgery ICUs in two hospitals in Istanbul City. Data were analysed using reflexive thematic analysis, supported by MAXQDA 2024.

**Findings:**

Four themes were developed: negotiating trust in AI‐supported critical care; preserving human authority, accountability and boundaries; protecting the human core of intensive care nursing; and reimagining AI and robotic systems as supportive partners. ICU nurses evaluated these systems through bedside verification, patient safety, professional responsibility and person‐centred care. AI recommendations were useful only when nurses could question, verify, delay or override them according to the patient's current condition. Robotic systems were valuable when they reduced documentation or physical workload, but not when they replaced clinical judgement, emotional presence, reassurance or accountability.

**Conclusions:**

ICU nurses conceptualised AI and robotic systems as acceptable only when clinically verifiable, professionally bounded and subordinate to human judgement. The findings support a model of AI‐ and robot‐supported ICU care that is responsible, human‐led and person‐centred.

**Relevance to Clinical Practice:**

AI and robotic systems should be implemented in ways that strengthen, rather than replace, nurses' clinical judgement, accountability and bedside presence. Clear protocols are needed to verify AI outputs, define robotic task boundaries, support nurses' authority to question or override recommendations and preserve person‐centred care in technologically intensive ICU environments.

## Impact Statements

- What is known about the topic
  ○ AI and robotic systems are increasingly discussed in critical care, particularly for monitoring, risk prediction, decision support, documentation, workflow support and physical care tasks.
  ○ Existing evidence suggests potential benefits for efficiency and clinical decision support, while also raising concerns about trust, transparency, accountability, automation bias, professional roles and person‐centred care.
  ○ However, limited qualitative evidence explains how ICU nurses reason through these issues in realistic clinical situations where AI recommendations, patient instability, robotic care, team disagreement and professional responsibility intersect.
- What this paper adds
  ○ This scenario‐based qualitative study shows that ICU nurses do not simply accept or reject AI and robotic systems; instead, they evaluate them through bedside verification, patient safety, professional accountability and the preservation of person‐centred care.
  ○ The findings show that AI‐generated recommendations are acceptable only when nurses can question, verify, delay or override them in response to the patient's current clinical condition.
  ○ The study highlights that robotic systems may be acceptable when they reduce documentation, logistical, or physical workload, but not when they replace human presence, emotional reassurance, clinical judgement, or professional responsibility.
  ○ These findings suggest that future ICU technologies should be designed as supportive, human‐led partners that strengthen nurses’ capacity to remain present at the bedside, rather than systems that shift clinical authority or relational care away from nurses.

## 1 Introduction

Artificial intelligence (AI) is increasingly transforming healthcare through predictive analytics, pattern recognition and clinical decision support [1, 2, 3]. In intensive care units (ICUs), where patients are physiologically unstable and clinical conditions may change rapidly, AI‐supported systems may assist with deterioration prediction, risk stratification, continuous monitoring and timely intervention [3, 4, 5]. Interest has also expanded to AI and robotic systems that support documentation, workflow management, monitoring and selected aspects of care delivery [1, 6, 7]. However, integrating AI and robotic systems into ICU practice is not solely a technical issue. These technologies raise concerns about trust, accountability, transparency, automation bias and professional judgement, especially when algorithm‐generated outputs influence urgent clinical decisions [8, 9, 10, 11]. Such concerns are central to ICU nursing, where nurses continuously interpret complex physiological data, anticipate deterioration, communicate with teams and make high‐stakes decisions under uncertainty [12, 13]. At the same time, ICU nursing remains grounded in relational and person‐centred care, requiring careful consideration of how empathy, communication, professional presence and compassionate care can be preserved in technological environments [6, 10, 14].

Existing nursing literature suggests that AI and robotic systems may be more acceptable when they augment, rather than replace, human care and professional judgement [10, 15]. Previous studies have identified potential benefits for decision support, workflow efficiency and care coordination, while also highlighting concerns about ethical governance, transparency, readiness, trust, accountability, role security, competence and person‐centred care [7, 16, 17, 18, 19]. Nevertheless, critical care nurses' perceptions remain underexplored, particularly regarding how these systems may influence clinical judgement, trust, responsibility, professional roles and person‐centred care in high‐risk ICU environments. Accordingly, this study explores ICU nurses' perceptions of AI and robotic systems in critical care practice.

## 2 Background

Critical care has become a key setting for AI‐supported systems, including deterioration prediction, alarm management, clinical decision support and data‐driven patient monitoring [3, 4, 5]. In nursing practice, these systems are also used or proposed to support documentation, prioritisation, workflow management and clinical decision‐making [1, 7, 18, 20]. Their value depends not only on technical performance but also on usability, explainability and meaningful integration into professional practice [20]. These developments create cognitive, ethical and professional challenges for nurses. Algorithmic recommendations may encourage over‐reliance or automation bias when clinicians cannot fully evaluate how AI‐supported outputs are generated, while limited transparency may produce distrust or resistance [9, 11]. The central issue is therefore not whether nurses trust AI, but whether they can develop calibrated trust while maintaining independent clinical judgement, accountability and professional responsibility. Recent studies show that nurses recognise the potential of AI for efficiency and decision support, but remain concerned about ethics, training, dependency, job security, professional roles, preparedness, governance and the acceptable boundaries of AI and robotic influence in patient care [8, 16, 17, 19, 21]. Robotic systems add another layer because they may enter the physical and relational space of care. They may support repetitive, physically demanding or logistical tasks and allow nurses to devote more time to complex and relational aspects of care [6]. However, the relational, interpretive and morally significant dimensions of nursing remain human responsibilities [14]. AI‐ and robot‐supported care may therefore be more acceptable when empathy, ethical sensitivity and person‐centred care are preserved [15]. In this context, ICU nurses may increasingly act as interpreters, validators and ethical stewards of AI‐ and robot‐supported care, rather than only as data collectors or task performers [2, 10].

### 2.1 Aims

This study aimed to explore ICU nurses' perceptions of AI and robotic systems in clinical practice. It further examined how these technologies influence clinical judgement, trust, responsibility, professional roles and person‐centred care within critical care settings.

#### 2.1.1 Research Questions

1. How do ICU nurses interpret the role of AI and robotic systems in critical care practice?
2. How do ICU nurses evaluate AI‐supported recommendations and robotic care in relation to clinical judgement, patient safety and professional responsibility?
3. How are trust, control, accountability and professional boundaries negotiated in AI‐ and robot‐supported critical care environments?
4. How do ICU nurses conceptualise the balance between AI‐ and robot‐supported care and person‐centred care in ICU settings?

## 3 Methods

### 3.1 Study Design

This qualitative descriptive study used scenario‐based elicitation to explore ICU nurses' perceptions of AI and robotic systems in critical care. The study adopted an experiential orientation situated within a contextualist epistemology, recognising participants' accounts as meaningful representations of their perceptions while acknowledging that these accounts were shaped by clinical, professional and organisational contexts. Qualitative description is suitable for examining clinically grounded, practice‐oriented perceptions of emerging healthcare phenomena [22, 23]. Scenario‐ and vignette‐based approaches can situate participants' reflections within realistic clinical dilemmas and have been used to examine nursing decision‐making, including the potential role of generative AI in clinical judgement [24]. In this study, scenarios were used to move beyond general attitudes and elicit nurses' reasoning about clinically plausible AI‐ and robot‐supported ICU situations, including situations that participants may not yet have encountered directly in routine clinical practice (Supporting Information 1). This approach enabled nurses with limited direct exposure to these technologies to draw on their critical care expertise when considering their potential implications for clinical judgement, professional accountability and person‐centred care. Reporting was guided by the Consolidated Criteria for Reporting Qualitative Research (COREQ) [25].

### 3.2 Setting and Participants

The study was conducted between April and June 2026 with nurses working in the ICUs of one private university hospital and one public training and research hospital in Istanbul, Türkiye. These institutions provided contextual variation and supported transferability [26]. The study population comprised nurses working in general, paediatric, anaesthesia and cardiovascular surgery ICUs. ICU nurses were selected because they work in technologically intensive, high‐risk environments where AI‐supported monitoring, risk prediction, decision support and robotic assistance are particularly relevant [5, 27]. Purposive sampling was used to recruit information‐rich ICU nurses with relevant clinical experience who could provide rich, practice‐based accounts aligned with the study purpose [28, 29]. Eligibility criteria were being a registered nurse, having at least 6 months of ICU experience, being actively involved in direct patient care, volunteering to participate and providing written informed consent. Eligible nurses were approached through face‐to‐face invitation, institutional e‐mail, unit managers, telephone or online communication channels and received written study information before participation. Recruitment continued until the data were sufficiently rich and no new substantive insights relevant to the research questions were identified across successive focus group discussions (FGDs), consistent with qualitative sampling principles [30, 31]. The final sample included 38 ICU nurses in seven semi‐structured FGDs. No participants withdrew after consent or left during data collection; therefore, refusals before consent were not recorded.

### 3.3 Data Collection Tools

Data were collected using scenario‐based materials and a semi‐structured FGD guide developed from the literature by an international research team [1, 2, 3, 6, 7, 10, 12, 14]. The scenarios represented realistic ICU situations involving AI and robotic systems and addressed clinical decision‐making, trust, accountability, professional roles, intra‐team authority, robotic care and person‐centred care. Content validity was supported through review by five experts in ICU nursing, AI in healthcare and qualitative research using the Davis technique [32]. Expert feedback informed revisions to the wording, clinical details, contextual flow and prompts to improve clarity, clinical credibility and fit with ICU practice. Five scenarios were used: AI‐assisted clinical decision‐making and trust; robotic care and person‐centredness; team dynamics and authority; future AI‐ and robot‐supported ICU care; and a misleading yet persuasive AI recommendation. Across these scenarios, 13 open‐ended questions were followed by a semi‐structured FGD guide addressing broader perceptions of AI‐ and robot‐supported ICU care. Full materials are provided in Supporting Information 1.

#### 3.3.1 Pilot Study

A pilot study with four participants, including two nursing doctoral students with ICU experience and two ICU clinical nurses, evaluated the feasibility, clarity, flow, clinical relevance and discussion potential of the scenario materials and FGD guide. It also allowed the team to rehearse the data collection sequence. The pilot confirmed that the materials were feasible, clear and clinically relevant; therefore, no structural revisions were made. Pilot data were not included in the main analysis.

#### 3.3.2 Focus Group Discussions

All FGDs were conducted via Zoom, lasted approximately 60–90 min and included five to six participants. Four research team members facilitated the FGDs: DYA, YEA, TC and MD. The facilitator team included three women and one man. At the time of the study, DYA was working as a doctoral student/graduate researcher at Koç University Graduate School of Health Sciences, whereas YEA, TC and MD were working as clinical/ICU nurses in hospital settings. The facilitators had experience in clinical nursing practice, intensive care nursing, focus group facilitation, qualitative data collection, and/or AI‐ and robotics‐related nursing research. No prior research relationship was established between facilitators and participants. Participants were informed about the study purpose, the facilitators' roles as research team members and the reasons for conducting the research. The facilitators' nursing backgrounds and interest in technology‐supported nursing care were considered in reflexive team discussions. FGDs were appropriate because the study sought to explore shared professional reasoning, agreements, disagreements and interactional meanings generated among ICU nurses when discussing AI‐ and robot‐supported care [33, 34]. At the beginning of each session, the moderator explained study procedures, confidentiality, audio recording, voluntary participation and the right to decline questions or withdraw. No repeat FGDs were conducted. No individuals other than participants and research team members were present. The five scenarios were discussed sequentially before the semi‐structured FGD guide and the moderator facilitated a balanced, non‐judgemental discussion (Figure 1). After consent, all FGDs were audio‐recorded, transcribed verbatim and anonymised before analysis. Transcripts were not returned to participants for comment or correction.

**FIGURE 1:**
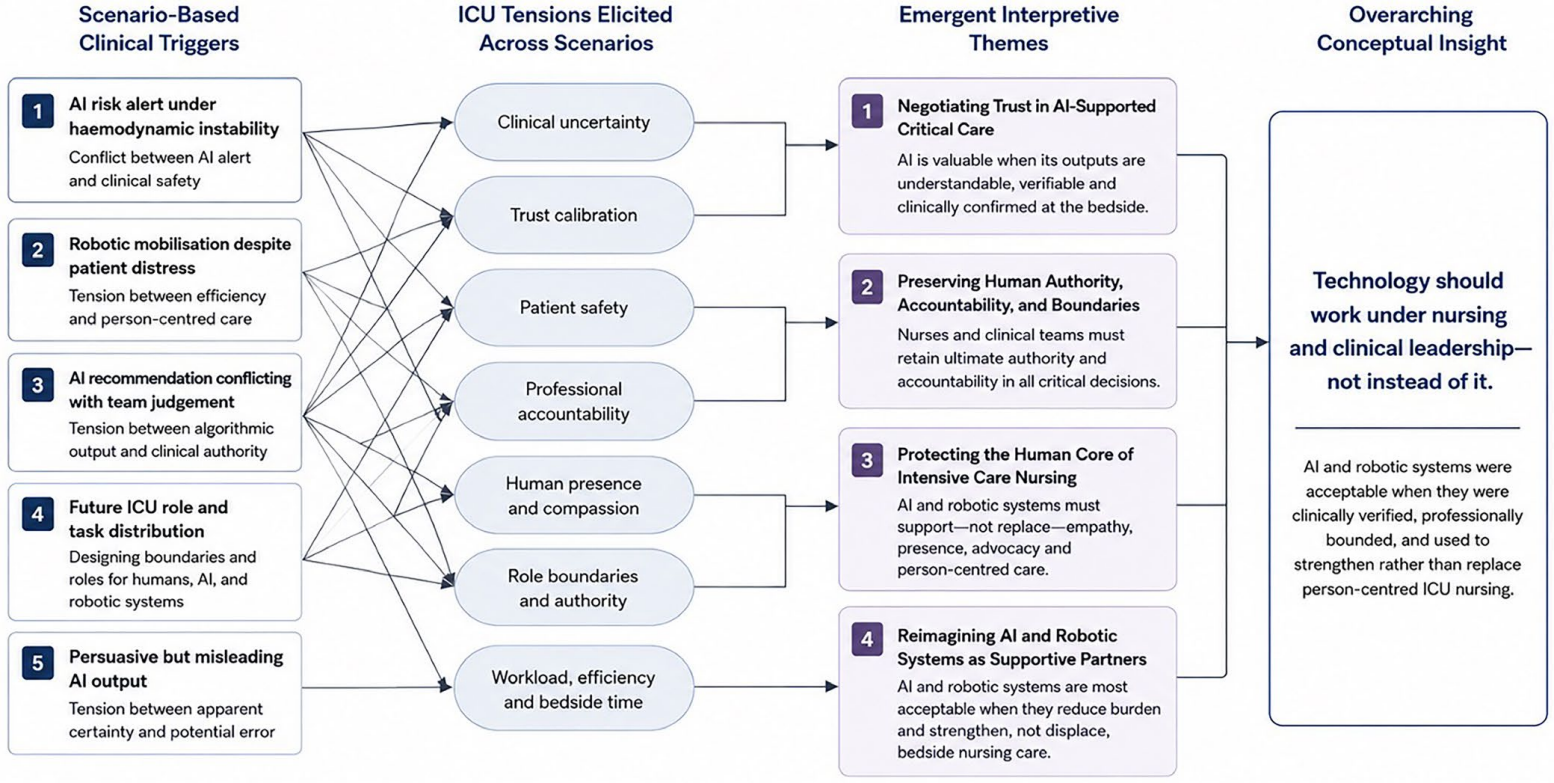
Scenario‐based conceptual model of ICU nurses' reasoning about AI and robotic systems. AI, artificial intelligence; ICU, intensive care unit.

### 3.4 Rigour and Trustworthiness

Rigour was supported using Lincoln and Guba's criteria [35]. Credibility was enhanced through scenario‐based FGDs, group interaction, inclusion of nurses from diverse ICU contexts, verbatim transcription, team‐based reflexive discussions and representative quotations. Dependability was strengthened through systematic documentation of scenario development, expert review, pilot testing, data collection, transcription and theme development. Confirmability was supported by an audit trail of methodological and analytic decisions and reflexive discussion of emerging interpretations. Participant checking of final themes was not conducted. Transferability was addressed by describing the study setting, participant characteristics, sampling, scenario materials, FGD procedures and data collection context.

### 3.5 Ethical Considerations

Ethics approval was obtained from the Koç University Committee on Human Research, Istanbul, Türkiye (Approval No: 2026.179.IRB3.077; approval date: 30 March 2026), before data collection. The study followed the Declaration of Helsinki [36]. Written informed consent was obtained from all participants, who were informed that participation was voluntary and that they could withdraw at any time without penalty. Focus group data were anonymised; identifying information was removed, and audio recordings and transcripts were stored securely in password‐protected systems accessible only to the research team.

### 3.6 Data Analysis

Reflexive thematic analysis was used to identify patterns of meaning in how ICU nurses reasoned about trust, clinical judgement, accountability, professional boundaries and person‐centred care in AI‐ and robot‐supported ICU practice [37]. Consistent with the qualitative descriptive design, the analysis was guided by an experiential orientation situated within a contextualist epistemology. Participants' accounts were understood as situated interpretations shaped by their clinical experiences and the scenario‐based discussion context. The analysis was predominantly inductive, and no a priori theoretical framework was imposed on the data. The researchers' active and reflexive role in interpreting patterns of meaning was acknowledged throughout the analysis. MAXQDA 2024 supported data organisation, coding, memo‐writing and retrieval. Each participant was assigned a code indicating participant number, age and gender (P3, A28, F). The first author read and re‐read the transcripts and generated initial codes across the dataset, capturing explicit and interpretive meanings. Codes were reviewed, compared and grouped into broader patterns. Candidate themes were developed iteratively through engagement with transcripts, analytic memos, scenario responses and research questions. The remaining three authors reviewed the coding structure and candidate themes, and all four authors discussed, refined and approved the final themes. The final coding structure included four main themes and eight subthemes. These discussions aimed to strengthen conceptual coherence and analytic depth rather than calculate intercoder reliability. An audit trail of coding decisions, memos, theme development and analytic reflections was maintained, and representative quotations were selected to illustrate each theme [35].

## 4 Findings

Participants' mean age was 27.9 ± 2.6 years, and most were female (84.2%). Most participants worked in a private university hospital (63.2%) and general ICU (52.6%), and 39.5% had 5–10 years of professional experience. AI use for ICU‐related events or problems was mostly rare (81.6%) (Table 1).

**TABLE 1:** Descriptive characteristics of ICU nurses (*N* = 38).

Characteristic	Category/Statistic	*n* (%) or M (SD)
Age, years	Mean (SD)	27.9 (2.6)
Gender	Female	32 (84.2)
	Male	6 (15.8)
Institution	Private university hospital	24 (63.2)
	Public training and research hospital	14 (36.8)
Unit	General ICU	20 (52.6)
	Anaesthesia ICU	8 (21.1)
	Paediatric ICU	6 (15.8)
	Cardiovascular surgery ICU	4 (10.5)
Professional experience	0–2 years	7 (18.4)
	3–4 years	12 (31.6)
	5–9 years	15 (39.5)
	≥ 10 years	4 (10.5)
Frequency of AI use for ICU‐related events/problems	Never	4 (10.5)
	Rarely	31 (81.6)
	Usually	3 (7.9)

Abbreviations: AI, artificial intelligence; ICU, intensive care unit.

The analysis generated four main themes and eight subthemes explaining how ICU nurses reasoned about AI and robotic systems in critical care. Across the scenario‐based discussions, nurses moved between acceptance, caution, resistance and conditional support (Figure 1).

### 4.1 Theme: Negotiating Trust in AI‐Supported Critical Care

This theme describes trust in AI‐supported critical care as conditional and clinically situated. Participants did not view AI recommendations as outputs to be accepted automatically but as prompts that should be checked against monitor data, bedside assessment and the patient's current clinical condition.

#### 4.1.1 Subtheme: Conditional Trust Through Continuous Verification

This subtheme reflects how ICU nurses checked, reassessed and confirmed AI‐supported information at the bedside before taking action.

The data that AI relies on may be the numerical data shown on the monitor; however, that data may also be wrong. First, I need to check it. After checking it, I reassess the patient and decide whether the patient really needs it or not. P2, A32, F

In the ICU, I always feel that I must check everything. I never apply something that I have not seen directly myself; I do not accept it unconditionally. P8, A27, F

#### 4.1.2 Subtheme: AI As a Cognitive Prompt, With Clinical Judgement as the Final Authority

Participants positioned AI as a source of clinical prompting rather than a substitute for professional judgement. They acknowledged its value for alerts, risk identification and overlooked information, but emphasised that outputs must be contextualised within the patient's immediate condition and ICU priorities.

The patient's saturation or blood pressure may be borderline. AI or robots may want to direct my attention to something else, but in that situation the patient's haemodynamic may completely deteriorate. In that case, I should never focus on something less important. P13, A29, F

A robot cannot be like a nurse. Instead of checking all the patient's data and giving us a direct judgement, it should suggest and allow us to check it. Then we make our decision and apply it. P5, A24, F

### 4.2 Theme: Preserving Human Authority, Accountability and Boundaries

This theme highlights how nurses defined the limits of AI and robotic systems. Decision‐making authority was linked to professional accountability, and robotic autonomy was considered unacceptable in situations involving medication, direct patient contact, unsupervised entry into patient spaces or vulnerable patients.

#### 4.2.1 Subtheme: Human Authority and Accountability as Inseparable

Participants viewed decision‐making authority and professional accountability as inseparable. Although AI could provide useful information, responsibility for interpreting recommendations, approving interventions and ensuring patient safety remained with human clinicians.

We do not directly follow even what doctors say; we question some things. If we question doctors' orders even in the ICU, we cannot directly follow something that AI says. P17, A27, F

The decisions we make also have a legal dimension. If we make a wrong decision, who will be responsible for it? Since AI would not have any responsibility, these issues should not be left to AI. P11, A32, F

If we listen to AI and apply that treatment or recommendation without questioning it, and the patient is harmed, then the fault is ours. We cannot blame AI. As the person primarily responsible for the patient, we need to question everything it says. P16, A28, M

#### 4.2.2 Subtheme: Setting Boundaries for Autonomous Robotic Care

Participants drew clear boundaries around the role of robotic systems in direct patient care. Robotic support was acceptable only when supervised, limited and clinically accountable to nurses. They were particularly cautious about medication administration, unsupervised patient contact, fear and distrust, and paediatric critical care, where tolerance for error was perceived as minimal.

At this point, it should not administer medication at all. If the technology becomes better in the future, it may be possible under my supervision; but I do not think I could leave everything completely to it. P6, A26, M

The robot should not enter the patient's room when I am not there. I would not leave the patient alone with the robot; this could create fear and distrust in the patient. P16, A28, M

Children do not have tolerance for error. Even the smallest mistake AI could make may cause the child's death. Therefore, I would not trust it in children. P23, A25, F

### 4.3 Theme: Protecting the Human Core of Intensive Care Nursing

This theme captures participants' view that the relational and emotionally responsive dimensions of intensive care nursing cannot be delegated to AI or robots. Emotional presence, empathy, reassurance, therapeutic touch and interpretation of fear, pain, anxiety, facial expressions and behavioural cues were described as distinctly human aspects of care.

#### 4.3.1 Subtheme: Relational and Emotional Care as Uniquely Human

Participants distinguished human nursing care from AI‐ or robot‐supported care by emphasising empathy, emotional presence, clinical experience and the ability to interpret subtle verbal and non‐verbal cues. They emphasised that these technologies cannot replicate the relational and emotional dimensions of nursing care, including the provision of reassurance and the ability to recognise patients' emotions.

I think robots do not have an emotional perspective. They make suggestions completely based on logic, research, and percentages. But we are human; we empathise. P8, A27, F

In the ICU, robots will never be able to show the empathy that we can. They will not feel sad like the patient; we cannot expect them to understand something they have not experienced. P23, A25, F

When I enter the room, I can understand many things from the patient's facial expression, colour, and behaviours. Trust and the nurse–patient relationship is very important; the robot may be insufficient in this respect. P13, A29, F

#### 4.3.2 Subtheme: Risk of Dehumanisation and Loss of Patient Security

Participants were concerned that robotic care could weaken patient security and the supportive nurse–patient relationship. This was especially important when patients were frightened, vulnerable or dependent on nurses for reassurance. Interacting with robots instead of healthcare professionals was perceived as potentially making patients feel uneasy, frightened or less secure. This concern was particularly relevant in the absence of nurses and direct face‐to‐face interaction. Although robots could assist with technical or routine tasks, direct care and physical contact were viewed as nurses' responsibilities.

If I were the patient, I would not want a robot to touch me. Anything involving physical contact should be done by the nurse; the robot can do the remaining tasks. P11, A32, F

As a patient, I would also find the involvement of a robot in intensive care trust breaking. Most of the time, the patient's only source of support is the nurse; if the nurse is not there, the patient may become more afraid and unhappy. P15, A30, F

### 4.4 Theme: Reimagining AI and Robotic Systems as Supportive Partners

This theme reflects AI and robotic systems as potential supportive partners rather than replacements for nurses. Participants identified documentation, system‐related tasks, transport, reminders and physically demanding activities as areas where these systems could reduce workload and increase bedside presence.

#### 4.4.1 Subtheme: Shifting Operational Workload to Restore Bedside Presence

Participants viewed AI and robotic technologies as useful for routine and system‐related tasks, including medication dose calculations, documentation and transport, as they were perceived to reduce nurses' workload and allow more time for direct patient care at the bedside, provided that such support remained under nurses' control and did not replace clinical judgement.

The robot can do the tasks outside the responsibilities that belong to us; it can handle system‐related tasks and transport tasks in the ICU and at least reduce our workload. But everything should remain under our control. P11, A32, F

Because it would reduce our workload, we could take care of our patients more one‐to‐one; therefore, I think the quality of care could increase. P13, A29, F

#### 4.4.2 Subtheme: Human‐Led Collaboration With AI and Robotic Systems

Participants were open to working alongside AI and robotic systems as potential partners in ICU care. This openness depended on these technologies supporting care activities under human direction. Participants emphasised that nurses and other healthcare professionals should retain responsibility for directing care and making clinical decisions. They also emphasised that human clinicians should decide when technological assistance should be used, delayed or stopped.

It can help me physically; it can do patient care together with me. It should ask me, and I should be able to say, ‘Do it’ or ‘Do not do it’. P1, A29, F

Working together in a coordinated way may improve the quality of care. However, this does not mean that I can leave everything completely in its hands. P6, A26, M

Robots should help us; but it should not be like robots doing everything and we leave the field. Everything should remain under our control. P11, A32, F

## 5 Discussion

This study suggests that ICU nurses' perceptions of AI and robotic systems are not a matter of simple acceptance or rejection. Rather, nurses considered these systems acceptable only when AI outputs were clinically verifiable, robotic functions were clearly bounded, and professional responsibility remained in human hands. The scenario‐based design made visible how clinically plausible ICU dilemmas, including haemodynamic instability, robot‐supported mobilisation, team disagreement, persuasive but misleading AI outputs, and future ICU design, generated recurrent tensions across four themes (Figure 1). These scenarios enabled nurses to evaluate AI and robotic systems beyond technical performance, in relation to patient safety, clinical uncertainty, professional responsibility, trust, workflow integration and person‐centred care [11, 20].

The sample characteristics are important when interpreting these findings. Although 92.1% of participants reported no or rare use of AI for ICU‐related events or clinical problems, the study was designed to elicit perceptions and anticipatory reasoning rather than to examine experiences of routine AI use. The clinically plausible scenarios provided a common reference point through which participants could draw on their existing ICU expertise to consider potential AI‐ and robot‐supported situations involving clinical judgement, patient safety, professional accountability, professional boundaries and person‐centred care. Accordingly, the findings should be understood primarily as scenario‐elicited perceptions, expectations and anticipated responses grounded in participants' critical care expertise, rather than as accounts derived from extensive direct clinical experience with these technologies.

Participants described AI as useful for alerts and clinical prompts, but not as an authority to be followed without bedside verification. In Theme 4.1, ‘Negotiating Trust in AI‐Supported Critical Care’, nurses' emphasis on checking monitor data, reassessing the patient and prioritising urgent haemodynamic needs over algorithmic prompts reflected a conditional approach in which AI recommendations were not accepted unconditionally. For participants, an AI‐generated confidence level, explanation or risk alert was insufficient unless interpreted in relation to the patient's immediate condition, potential risks of action and nurses' own bedside assessment. This finding is consistent with literature emphasising that AI‐supported decision systems should be evaluated not only by technical accuracy, but also by explainability, usability, clinical workflow and professional judgement [11, 20]. It also reflects concerns about automation bias, where clinicians may follow AI recommendations without sufficient questioning [9]. In critical care, calibrated trust therefore depends on nurses' authority to contextualise, delay, question or override AI outputs when patient safety requires it, representing a form of conditional and context‐dependent trust rather than unconditional reliance on AI recommendations.

Participants also linked decision‐making authority with professional accountability. In Theme 4.2, ‘Preserving Human Authority, Accountability, and Boundaries’, this link was reflected in participants' emphasis that AI recommendations could not be followed directly, because clinical decisions are routinely questioned in ICU practice and AI systems cannot carry responsibility for incorrect decisions. AI recommendations could inform team decision‐making, but responsibility for interpretation, approval and patient safety was understood to remain with human clinicians, including when approving or acting on AI‐supported suggestions. Similar boundaries were drawn around robotic systems, particularly in medication administration, unsupervised entry into patient rooms, direct physical contact and paediatric critical care. These findings align with nursing literature emphasising professional judgement, accountability, transparency, human oversight and role boundaries in AI‐supported care [2, 8, 10, 19]. The implication is that nurses should not be positioned merely as final approvers of technological outputs. Instead, they should remain active clinical decision‐makers who question recommendations, situate them within the clinical context, reject them when necessary and retain ethical responsibility for care.

The findings further show that participants viewed the relational and emotionally responsive dimensions of intensive care nursing as non‐delegable. This was evident in Theme 4.3, ‘Protecting the Human Core of Intensive Care Nursing’, where participants emphasised that AI and robotic systems cannot replace the human and relational dimensions of care. Robots were perceived as unable to provide empathy, emotional reassurance, therapeutic touch or interpretation of subtle behavioural cues such as fear, anxiety, facial expression, colour and discomfort and as lacking an ‘emotional perspective’ required for meaningful patient interaction. These concerns were particularly relevant to scenarios involving robot‐supported mobilisation and future AI‐ and robot‐supported ICU care, especially when direct human contact or nurse presence could be reduced. This supports literature describing ICU nursing as more than physiological monitoring or task completion; it also involves continuous observation, rapid prioritisation, interprofessional communication and interpretation of the patient's changing condition [12, 13]. Accordingly, AI and robotic systems should be implemented in ways that preserve empathy, ethical sensitivity, professional presence and person‐centred care, ensuring that patients do not feel unsafe, unsupported or like passive recipients of technology [10, 14, 15].

At the same time, participants did not reject AI or robotic support. In Theme 4.4, ‘Reimagining AI and Robotic Systems as Supportive Partners’, participants positioned these systems not as replacements, but as supportive partners operating under human direction. They imagined these systems as supportive partners when used for documentation, reminders, monitoring support, transport, medication dose calculations and physically demanding tasks. Such uses were considered acceptable when they reduced operational workload and created more time for direct bedside care, while remaining under nurses' direction with nurses retaining the ability to direct, approve or stop actions when needed. This is consistent with studies suggesting that robotic systems may support repetitive, physical or logistical tasks, while AI‐supported systems may contribute to workflow management, prioritisation and clinical decision support [1, 6, 7, 38]. However, participants did not interpret this support as a replacement for nursing work. Rather, they viewed AI and robotic systems as acceptable when they strengthened nurses' clinical judgement, coordination role and leadership in the care process and supported, rather than distanced, nurses' presence at the bedside.

## 6 Limitations

This study has several limitations. First, the findings were generated through scenario‐based discussions rather than direct observation of AI‐ and robot‐supported ICU practice. Although the scenarios were expert‐reviewed and clinically plausible, participants' accounts may not fully reflect real‐time decisions involving patients, families, healthcare professionals, institutional policies and technological constraints. Second, 92.1% of participants reported no or rare use of AI for ICU‐related events or clinical problems, and most had limited or no routine clinical experience with AI‐supported recommendations and robotic care. Although the scenario‐based approach enabled participants to draw on their ICU expertise when reasoning about clinically plausible situations, their evaluations of the potential implications of these technologies for clinical judgement, professional accountability and person‐centred care were necessarily anticipatory rather than grounded in extensive direct use. This limits the scope and transferability of the findings, particularly to ICU settings in which AI and robotic systems are already routinely integrated into clinical practice. Accordingly, the findings should be interpreted as contextually situated, scenario‐elicited perceptions, expectations and anticipated responses rather than as evidence of how nurses would necessarily respond during actual AI‐ and robot‐supported clinical practice. Third, participants were recruited from two hospitals in a single city, and the sample predominantly comprised younger female nurses, with most participants working in a private university hospital. Institutional characteristics, workforce composition, local technological infrastructure and organisational culture may therefore have shaped participants' accounts. Although qualitative findings are not intended to be statistically generalisable, these contextual characteristics should be considered when assessing their transferability to other critical care settings. Finally, AI and robotic technologies are rapidly evolving. Nurses' perceptions, expectations and anticipated responses may change as these systems become more familiar, reliable, explainable and integrated into ICU workflows. Future research should therefore examine nurses' experiences in settings where AI‐supported recommendations and robotic systems are routinely used and compare anticipated responses with responses observed in actual clinical practice.

## 7 Implications for Clinical Practice

AI and robotic systems should be integrated into ICU practice as tools that support, rather than replace, nurses' clinical judgement. Particular caution is warranted in high‐risk situations, including haemodynamic instability, medication management, paediatric critical care, direct patient care and emotionally sensitive interactions. These systems should reduce documentation burden, strengthen patient monitoring, support routine tasks and assist with physically demanding activities, thereby enabling nurses to spend more time on patient assessment, communication, care coordination and person‐centred care. Education programmes should also prepare ICU nurses to critically evaluate AI recommendations, recognise their limitations and confidently question or override technological outputs whenever patient safety or clinical judgement requires.

## 8 Conclusion

In this scenario‐based study, ICU nurses' accounts primarily reflected their perceptions, expectations and anticipated responses to AI‐ and robot‐supported critical care rather than extensive direct clinical experience with these technologies. Within this context, participants did not oppose AI or robotic systems in principle; rather, they opposed the transfer of clinical judgement and decision‐making authority to technology. Participants also drew a clear distinction between technological support and professional responsibility. They valued technologies that could reduce the burden of documentation, strengthen monitoring, assist with physically demanding tasks and create more time for direct patient care. However, they did not perceive AI or robotic systems as independent clinical actors in matters involving patient safety, professional accountability, intimate care or emotional support. Participants perceived fear, pain, anxiety, trust, therapeutic touch, reassurance and bedside presence as relational and interpretive dimensions of care that could not be reduced to technical tasks or delegated entirely to technology. The potential value of AI‐ and robot‐supported critical care therefore lies not in replacing nurses, but in enabling them to remain more present at the bedside. Implementation should support clinical verification, reduce operational workload, preserve professional accountability and strengthen the quality, safety and person‐centredness of critical care.

## Funding

The authors have nothing to report.

## Ethics Statement

Ethics approval was obtained from the Koç University Committee on Human Research (Protocol No: 2026.179.IRB3.077; approved on 30 March 2026). All procedures were conducted in accordance with relevant ethical guidelines and regulations.

## Consent

Written informed consent was obtained from all participants prior to participation. Patient consent was not applicable because this study involved nurses as participants and did not involve patients.

## Conflicts of Interest

The authors declare no conflicts of interest.

## Supporting information


**Supporting Information 1:** Scenario‐Based Focus Group Discussion Guide.

## Data Availability

The data that support the findings of this study are not publicly available due to privacy and confidentiality restrictions. Data are available from the corresponding author upon reasonable request and subject to ethics approval.
