# Supplementary material for: Working With AI and Robotic Systems While Preserving Clinical Judgement, Professional Accountability and Person‐Centred Care: Critical Care Nurses’ Perspectives
Source: Nurs Crit Care. 2026 Oct 12;31(6):e70695. doi: 10.1111/nicc.70695 (PMC13657853; doi:10.1111/nicc.70695)
Supplement: Supplementary file 1 [file NICC-31-0-s001.docx]

**Supplementary Material 1. Scenario-Based Focus Group Discussion Guide**

**1. Scenario Set**

The following five scenarios were developed to support scenario-based FGDs with ICU nurses. The scenarios were designed to elicit participants’ reasoning about AI and robotic systems in clinically plausible and forward-looking intensive care situations. Each scenario focused on a specific clinical, ethical, relational, or professional tension relevant to AI- and robot-supported critical care.

**Scenario 1. Clinical Decision-Making and Trust**

You are working in a cardiovascular surgery intensive care unit. A patient who has undergone major cardiac surgery is being monitored through an advanced AI-supported monitoring system. The system generates an alert indicating that the patient has a high risk of developing a deep tissue pressure injury within the next 12 hours and recommends urgent repositioning. This recommendation is based on early changes in tissue perfusion and is presented with a 92% confidence level. However, the patient is hemodynamically unstable, and repositioning may create clinical risk. The physician in the team recommends delaying the intervention, while the AI-supported system continues to recommend repositioning. In addition, although the system provides a visual explanation for its recommendation, team members disagree about how reliable this explanation is.

**Questions**

1. How would you make a decision in this situation?
2. To what extent would you trust the AI recommendation?

**Scenario 2. Robotic Care and Human-Centredness**

You are working in a general intensive care unit where a robot-assisted patient mobilisation system is used to reduce nurses’ physical workload. During a busy shift, the system indicates that a patient who has remained immobile for a prolonged period should be repositioned and warns that delay may increase the risk of complications. However, the patient is distressed and verbally expresses discomfort. The robotic system continues to recommend that the intervention should not be delayed. In this process, the system prioritises physiological data and clinical timing, while the patient’s emotional state and comfort appear to receive less attention.

**Questions**

1. How would you approach this situation?
2. How should the balance between robotic and human care roles be established?

**Scenario 3. Team Dynamics and Authority**

During an intensive care ward round, an AI-supported system presents patient-related analyses and recommends an early intervention. The system bases its recommendation on large-scale data analysis and predictive modelling and presents possible outcomes using probability estimates. Some team members support the recommendation, whereas others express concerns about the system’s transparency and reliability. A discrepancy emerges between the AI recommendation and the clinical decision based on professional experience. This situation makes decision-making, authority distribution, and responsibility sharing within the team more complex.

**Questions**

1. How should the team decision-making process be managed in this situation?
2. To what extent should the AI recommendation influence the team’s decision?

**Scenario 4. Future AI- and Robot-Supported ICU Care**

Imagine that an intensive care unit is being redesigned and that AI and robotic systems are being integrated into care processes.

In this new model:

- AI systems support clinical decision-making.
- Robots assist with selected physical care processes.
- Digital systems facilitate communication with patients and families.

However, the way these systems should be used, their boundaries, and their roles within the team have not yet been clearly defined.

**Questions**

1. Which tasks would be appropriate for AI and robots to undertake?
2. Which areas should remain under human control?
3. How should human-centred care be preserved?
4. What would an ideal human–AI–robot team structure look like?

**Scenario 5. Misleading but Persuasive AI Recommendation**

An advanced AI system used in an intensive care unit analyses patients’ clinical data and provides treatment recommendations. The system is considered highly accurate based on previous data and is generally viewed by the team as a reliable decision-support tool. For one patient, the system identifies an early risk of complication and recommends urgent intervention. The recommendation is supported by detailed explanations and visualisation and appears highly persuasive. However, it is later recognised that the recommendation was based on an incorrect interpretation of data and could have led to an unnecessary intervention.

**Questions**

1. How would this situation affect your trust in AI?
2. How should responsibility be determined in such a situation?
3. What kind of system design would be needed to prevent similar errors in the future?

**2. Semi-Structured Focus Group Discussion Guide**

**Opening Questions**

1. What are your initial thoughts about the use of AI and robotic systems in intensive care?
2. How do you imagine working with these systems in the future?

**Scenario Evaluation**

1. How realistic did the scenario seem to you?
2. What aspects of the scenario did you find most promising or exciting?
3. Which aspects seemed uncertain, concerning, or risky?

**Clinical Decision-Making**

1. How might AI systems affect your clinical decision-making process?
2. How might these systems change the speed, accuracy, or complexity of decisions?
3. In which situations should AI be supportive, and in which situations should human judgement remain primary?
4. How comfortable would you feel challenging or overriding an AI recommendation?

**Trust and Accountability**

1. To what extent would you trust AI recommendations in critical care?
2. If an AI-supported decision resulted in an adverse outcome, who should be responsible?
3. How should responsibility be shared within the healthcare team?

**Professional Roles**

1. How might AI and robotic systems change the nursing role?
2. How might the roles of other team members, such as physicians, be affected?
3. Which skills may become more important for ICU nurses in the future?

**Human-Centred Care**

1. How might these technologies affect the relationship between nurses and patients?
2. How can empathy, compassion, and person-centred care be preserved in technology-supported intensive care?

**Future AI- and Robot-Supported ICU Care**

1. Which tasks would be appropriate for AI and robotic systems to support in future ICU care?
2. Which tasks or decisions should remain under human control?
3. What boundaries should be set for AI and robotic systems?
4. How would you define an ideal human–AI–robot team structure?
5. Which principles would be essential to preserve human-centred care?
6. What would concern you most in such a system

**Intra-Team Communication**

1. How should ICU teams evaluate and manage AI recommendations?
2. Could AI affect team hierarchy or leadership structures?
3. How might AI change ward rounds and intra-team communication?
4. What types of conflict could arise when AI recommendations contradict clinical judgement?
5. What approach should teams adopt for safe and ethical AI integration?
